# Virtual reality videos for training and protocol dissemination during a pandemic

**DOI:** 10.15694/mep.2020.000140.1

**Published:** 2020-06-30

**Authors:** Glenn Posner, Jerry Maniate, Jennifer Dale-Tam, Kaitlin Endres, Janet Corral

**Affiliations:** 1 The Ottawa Hospital & The University of Ottawa; 2 The Ottawa Hospital; 3 University of Arizona Tucson

**Keywords:** Simulation, Virtual Reality, Pandemic

## Abstract

This article was migrated. The article was marked as recommended.

Preparations for the COVID-19 pandemic required healthcare teams to practice known skills, such as intubation, with renewed consideration for safety, as well as develop new Standard Operating Procedures (SOPs) for health care delivery. In these conditions, translational simulation based-education (SBE) is a well-known tool that supports health care teams to improve the system using design thinking methods such as walkthroughs and team-based simulation. However, the pandemic has introduced two stressors on translational SBE simultaneously. Firstly, the need for rapid upskilling of front-line staff and rapid change to SOPs. Secondly, the need for social or physical distancing at work, such that it quickly became inappropriate for large groups of individuals to practice *in-situ* SBE and debrief together in close proximity. An educational approach that brings the best of translational SBE while minimizing contact and maximizing experiential learning is needed.

Digital learning has been rapidly adopted by much of medical education during the pandemic. Focusing on a strong alignment between learning goals with intended clinical performance change outcomes we sought to leverage a digital education format that allowed for low barriers to adoption, yet supported the experiential, dynamic reality of translational SBE. In the absence of the ability to quickly train large numbers of people due to the need for social distancing, an immersive experience that can only be provided by virtual reality (VR) videos was the next best thing. VR, using 360-degree video, supported the creation of instructional videos from SBE events in the hospital which allow the learner to immerse and explore multiple points within the scenario. We describe how the very act of recording a video assisted in the rapid development of SOPs through translational simulation. We then describe the use of VR to stay true to the spirit of simulation for experiential learning and nearly hands-on training.

## Introduction

Preparations for the COVID-19 pandemic required healthcare teams to practice known skills, such as intubation, with renewed consideration for safety, as well as develop new standard operating procedures (SOPs) for health care delivery (e.g. protected cardiac arrest management, transferring patients, cleaning rooms, where to resuscitate a newborn, etc.). In these conditions, translational simulation based-education (SBE) ( Brazil, 2017) is a well-known tool that supports health care teams to improve the system using design thinking methods such as walkthroughs and team-based simulation ( Petrosoniak *et al*., 2020). However, the pandemic has introduced two stressors on translational SBE simultaneously. Firstly, the need for rapid upskilling of front-line staff and rapid change to SOPs, which in the pandemic, may require same day response. Secondly, the need for social or physical distancing at work, such that it quickly became inappropriate for large groups of individuals to practice SBE and debrief together in close proximity. An educational approach that brings the best of translational SBE while minimizing contact and maximizing experiential learning is needed.

Digital learning has been rapidly adopted by much of medical education during the pandemic ( Kachra and Brown, 2020). Focusing on a strong alignment between learning goals with intended clinical performance change outcomes ( Biggs, 1996; Walsh, 2007) we sought to leverage a digital education format that allowed for low barriers to adoption, yet supported the experiential, dynamic reality of translational SBE. In the absence of the ability to quickly train large numbers of people due to the need for social distancing, an immersive experience that can only be provided by virtual reality (VR) videos was the next best thing. VR, using 360-degree video, supported the creation of instructional videos from SBE events in the hospital which allow the learner to immerse and explore multiple points within the scenario. We describe how the very act of recording a video assisted in the rapid development of SOPs through translational simulation. We then describe the use of VR to stay true to the spirit of simulation for experiential learning and nearly hands-on training.

## Description

A 360-degree 4K digital camera was used to capture video from exemplar in-situ simulation scenarios run by scenario authors and staff volunteers (physicians, resident physicians, nurses, respiratory therapists, medical radiation therapists, radiology technicians, and environmental service workers). Each video has an identified goal, specific learning objectives, and demonstrates an aspect of hospital preparedness for managing patients during the COVID-19 pandemic. Text overlays on key features within the videos were added in post-production, in order to tune learners’ attention to pivotal learning moments. In place of debriefing, participants are asked reflective questions at the end of the scenario, which they are encouraged to discuss with their team members and/or compare to evolving COVID-19 protocols.

**Figure 1.**
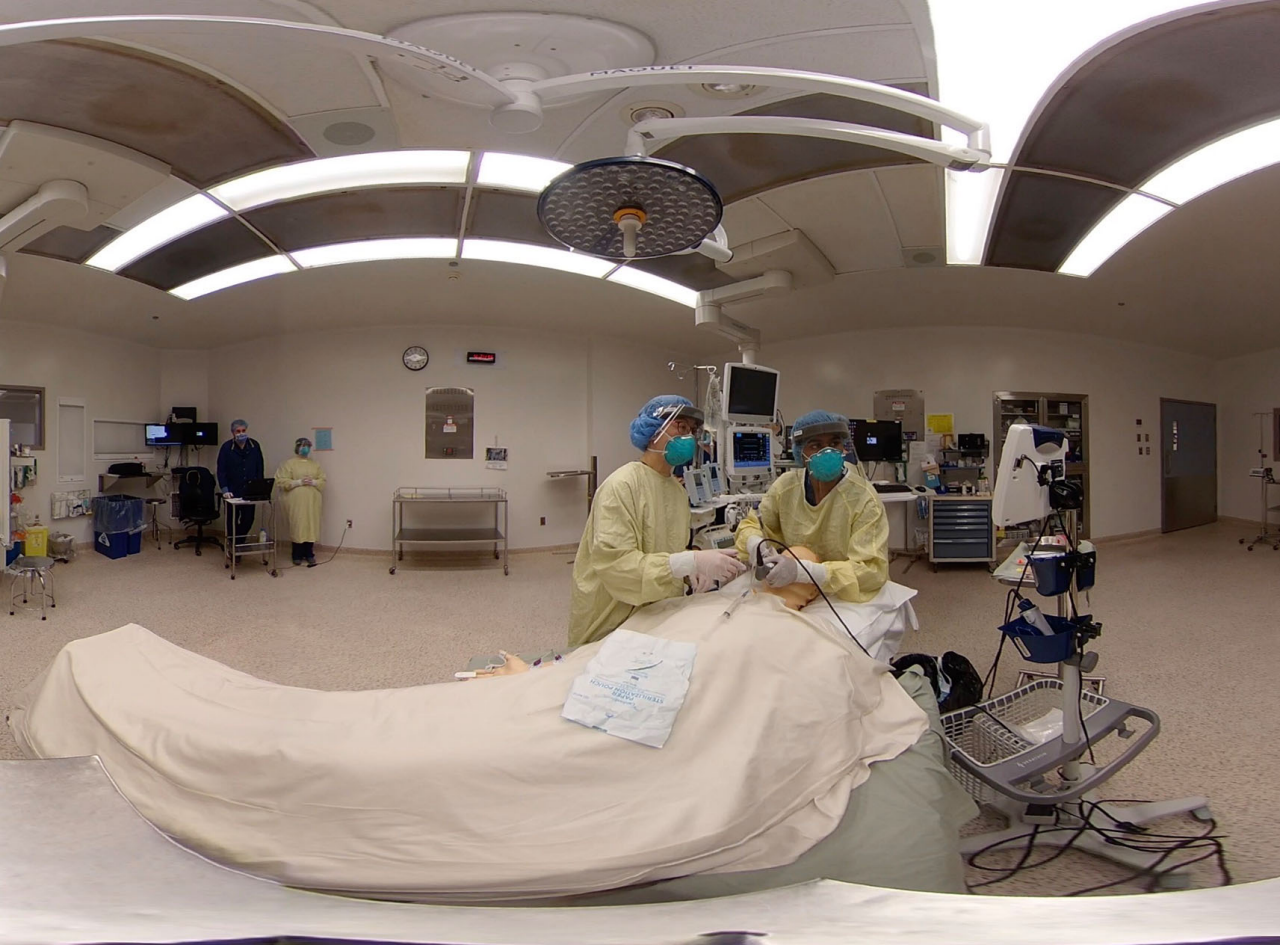
Suspected Covid-19 patient requires surgery

**Figure 2.**
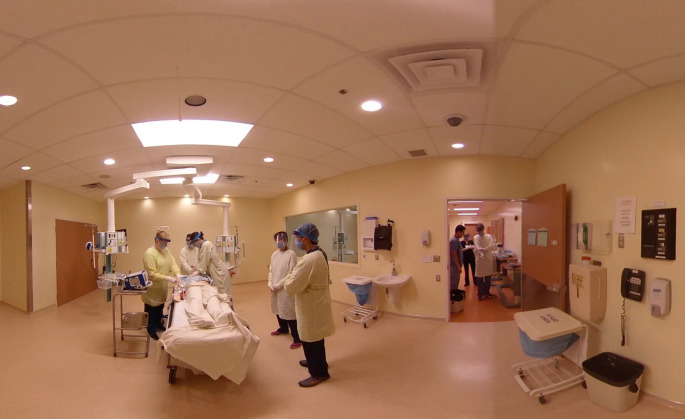
Managing Cardiac Arrest in the era of Covid-19

We were systematic in our approach to identifying training needs, and started in areas where sick patients would present first: the emergency department, the operating room ( Figure 1), the labour floor, and the medicine ward ( Figure 2). Next, we developed videos targeted at providers in diagnostic imaging, environmental services, and ambulatory care. Once we had covered all essential front-line areas of the hospital, we began to create content for patients and family caregivers. We created virtual tours of the hospital to manage expectations and allay some fears so patients would not be apprehensive to seek out the medical care they required. We created a PPE training video for the general public who may never have put on a mask before, to assist them when going into long-term care facilities or palliative care settings to provide care for their family members.

The videos were first hosted on a VR platform called WondaVR, but quickly moved to YouTube for reasons related to usability, democratization, and long-term access. The videos are best experienced using a VR headset but can be viewed on a computer by moving the image using a mouse, or on a gyroscope-enabled smartphone or tablet by moving the device or by moving the image with a finger. Videos were publicized within the organization via email to the relevant departments, and disseminated more broadly using twitter (@SPSP_TOH - TOH Simulation Patient Safety Program).

Creating videos began to drive the creation of new processes - the very act of recording an exemplar drove the need for the drafting or modifying of a standard operating procedure that was then tested in the actual clinical space using a mannequin and healthcare providers. Creating a SOP is often a long process involving many stakeholders, and often involves table-top exercises and “talking it out.” However, in the setting of a pandemic, the need to address system issues quickly and disseminate them via video-recording led to the use of simulated walkthroughs and practical development of SOPs to guide filming. The skills of simulation educators were used to anticipate problems and the act of recording a video served to ensure that new processes were logistically feasible.

## Potential Risks

Recording video at the hospital during a pandemic is not risk-free. While assembling a small group of individuals to record a durable video that can benefit many is preferable to bringing the same audience into the clinical environment, there is still risk of viral transmission between these participants. Honored by the professionalism and earnestness of our colleagues to disseminate new, potentially life-saving SOPs, we experienced an extraordinary response from colleagues who volunteered to be on health care teams in order to create these videos. We wore masks and practiced social distancing as much as possible between takes.

The other potential harm in preparedness training for a pandemic involves the use of a large amount of PPE for practice instead of real life. At a time when hospitals in Canada and around the globe are struggling to supply front-line healthcare workers with adequate protective equipment, how does a simulation program justify using copious amounts of PPE for a training video? We mitigated this risk in two ways, using simulation and reuse. During filming, we used simulated N95 masks and procedure masks manufactured in-house using everyday items as designed by medical students at the Northern Ontario School of Medicine in Sudbury ( Anderson, 2020). Face shields were cleaned with disinfectant wipes and reused. Cloth gowns were used wherever possible so that they could be laundered and reused.

## The Outcomes

To date, there have been over 12,000 views of our 19 clinical videos and nearly 10,000 views of our four patient-centred videos ( TOHEducation.ca, 2020) ( Table 1). In a society where the dissemination of protocols via email often leads to a lack of engagement (the “swipe-delete” phenomenon), the use of the VR modality has been able to provide a compelling vehicle for alerting healthcare providers to changes in policy and procedure. We hope that the clinical videos have allowed us to mentally prepare our healthcare colleagues locally and globally to deal with various patient scenarios related to COVID-19. Similarly, we hope that the virtual tours have helped us manage the expectations of our local patients and alleviate some of their anxiety about entering the hospital during a pandemic.

**Table 1.** Videos created in response to the pandemic, and the number of views of each on YouTube and WandaVR as of May 30, 2020.

Date posted	Title	Subject	Views
21-Mar	Suspected COVID-19 patient requires intubation in the ED	Emergency Medicine	2150
22-Mar	Simulation: Suspected Covid-19 patient requires surgery	Anesthesiology	556
22-Mar	In-situ Simulation of suspected Covid-19 pt deteriorating in the ER	Anesthesiology	609
28-Mar	Managing Cardiac Arrest in the era of Covid-19	Cardiac Arrest	3213
04-Apr	VR Tracheotomy on an awake COVID-19+ patient	Otolaryngology	343
04-Apr	VR Tracheotomy for Intubated Covid-19+ Patient	Otolaryngology	609
08-Apr	Safe Transfer of a Covid-19 Patient in/out of the CT Scanner	Radiology	535
11-Apr	Transesophageal Echocardiography for Covid-19+ Patients	Cardiology	140
11-Apr	Transfer to OR for Urgent Cesarean (Covid-19 era)	Obstetrics	1617
14-Apr	Environmental Services - Cleaning an ICU Room after COVID-19	Environmental Services	363
14-Apr	NICU1: Neonatal Resuscitation in the OR - Covid-19 Precautions	Neonatology	207
17-Apr	Endoscopy in the era of COVID-19	Gastroenterology	443
17-Apr	NICU3: Deterioration of baby in isolation room in NICU/SCN requiring urgent intubation (Covid-19)	Neonatology	217
18-Apr	NICU2: Neonatal Resuscitation adjacent to the OR - Covid-19 Precautions	Neonatology	158
21-Apr	Cardiac Arrest - Radiation Therapy Bunker - Covid-19	Cardiac Arrest	263
28-Apr	Caring for Patients with COVID-19: Nursing Workflow	Nursing	332
28-Apr	Ambulatory Care ENT during the COVID-19 pandemic	Otolaryngology	164
28-Apr	General Virtual Tour - Delivering at The Ottawa Hospital General Campus during a Pandemic	Obstetrics	4560
03-May	Civic Virtual Tour - Delivering at The Ottawa Hospital Civic Campus during a Pandemic	Obstetrics	4127
08-May	Personal Protective Equipment for Family Caregivers	Family Caregivers	303
13-May	Updated** - Managing Cardiac Arrest in the era of COVID-19	Cardiac Arrest	239
19-May	Personal Protective Equipment for Healthcare Workers in LTC	Long Term Care	31
22-May	Virtual Tour of TOH General Campus Emergency Room (COVID-19 pandemic)	Emergency Medicine	254
		Total	**21433**
		Total Clinical	**12189**
		Total Patient-Centred	**9244**

## Future Directions

Anyone with a 360-degree camera and appropriate video-editing software can adopt this innovation and adapt it to their context. As evidenced by the number of views, videos targeting patients and their families should be the next focus of projects like this. The COVID-19 pandemic has opened our eyes to how much we rely on in-person instructional activities in healthcare education. Now is the time to create medical school curricula and patient-experience materials that utilize VR and video in their instructional design to help prepare for the next pandemic.

## Take Home Messages

• During a pandemic, the need for social distancing makes training in groups a challenge; video recordings of exemplar scenarios in virtual reality can address this challenge.
• The act of creating a video drives the organization to rapidly develop new standard operating procedures and tests them in realtime.
• While watching a video is not the experiential learning that simulation educators typically espouse, an immersive virtual reality video is the best approximation of experiential learning that is appropriate during a pandemic.
• Recording videos during a pandemic is not ideal, due to the risks to healthcare providers and the potential for wastage of PPE, but these risks can be mitigated.

## Notes On Contributors

**Glenn Posner**, MDCM, M.Ed, FRCSC, is an Associate Professor in the Departments of Obstetrics and Gynecology and Innovation in Medical Education, University of Ottawa, and the Medical Director of the University of Ottawa Skills & Simulation Centre. ORCID: https://orcid.org/0000-0002-3375-8470

**Jerry Maniate**, MD, M.Ed, FRCPC, is an Associate Professor in the Departments of Medicine & Innovation in Medical Education, and Vice-President, Education for The Ottawa Hospital. ORCID: https://orcid.org/0000-0002-2112-4147

**Jennifer Dale-Tam,** RN, MSN, CCNC, is a nurse educator and simulation educator at The Ottawa Hospital. ORCID: https://orcid.org/0000-0003-4461-7216

**Kaitlin Endres**, B.Sc is a medical student at the University of Ottawa and a simulation technician at The Ottawa Hospital. ORCID: https://orcid.org/0000-0001-5797-5712

**Janet Corral**, MD, PhD, is Associate Dean of Curricular Affairs and Associate Professor in the Department of Medicine, College of Medicine, University of Arizona Tucson. ORCID: https://orcid.org/0000-0001-8576-6192

## Declarations

The author has declared that there are no conflicts of interest.

## Ethics Statement

Ethical approval was not required for this education method because it does not report research findings. Formal consent was sought from all of the participants in each photograph displayed in the manuscript, in writing.

## External Funding

This article has not had any External Funding
