# Supplementary material for: Virtual reality videos for training and protocol dissemination during a pandemic
Source: MedEdPublish (2016). 2020 Jun 30;9:140. [Version 1] doi: 10.15694/mep.2020.000140.1 (PMC10702642; doi:10.15694/mep.2020.000140.1)
Supplement: Peer review history

# Reviewer report: Reviewer response for version 1

**DOI:** 10.21956/mep.20117.r31028

**Published:** 2020-10-24

**Authors:** Ken Masters

**Affiliations:** 1 Sultan Qaboos University

This review has been migrated. The reviewer awarded 3 stars out of 5 An interesting paper on the use of virtual reality videos for training and protocol dissemination during a pandemic. The process appears to have been a wonderful learning and teaching experience; I also appreciate the fact that the videos have been made publicly visible.Two issues that I would like to see addressed:• The Abstract needs to be re-written. It appears to be a duplication of the Introduction, with a few minor edits. This is especially important because indexing systems show the Abstract by default.• There should be quite a bit of extra details about costs (e.g. time, human resources, financial cost, etc.), efforts, problems, etc. This is important, because readers who read the paper would be tempted to say, “Great, let’s do that!” without any real insight into the tremendous effort required to create these properly.• Please place a link to the videos – if not to the individual videos, then at least to the Channel so that it is easy for readers to find them. In the absence of a formal evaluation, this would be the best way for readers to see their value, and use them in their teaching.I look forward to Version 2 of this paper in which these issues are addressed.

Reviewer Expertise:

NA

No decision status is available

# Reviewer report: Reviewer response for version 1

**DOI:** 10.21956/mep.20117.r31029

**Published:** 2020-07-19

**Authors:** Daniel Kiely

**Affiliations:** 1 Hôpital du Centre-de-la-Mauricie

This review has been migrated. The reviewer awarded 4 stars out of 5 COMMENTS: Frank H. Netter, author of the renowned Atlas of Human Anatomy said, “The making of pictures is a stern discipline. One may ‘write around’ a subject where one is not quite sure of the details, but, with a brush in hand before the drawing board, one must be precise and realistic. The white paper before the artist demands the truth.” The same could be said for filming simulation videos. Those described here contain a wealth of detailed information which is useful in preparing healthcare teams for the COVID-19 pandemic. The article is well-written, to the point, and concise. The article is critically important in the current context where it is so important to prepare but, at the same time, our ability to use our conventional tools of face-to face simulation is limited. I have only one constructive suggestion. I found it somewhat difficult to view the videos on a cell phone using a 3G connection which is admittedly mostly my fault for not having a computer and a better internet connection at the time. However, I did think that it may have been worthwhile to film the scenarios simultaneously both with a 360 degree camera and a conventional camera because there may be some situations where it is easier to disseminate video filmed with a conventional camera. STRUCTURED RESPONSES TO QUESTIONS: Is the topic appropriate to medical education? Yes. Who are the target audience for this article and what could they expect to learn from it? All healthcare providers who may be called upon to care for patients with COVID-19 as well as the administrative staff who support them. Are the methods adequately described (to the point that, under ideal conditions, they could be re-produced)? YesAre the results displayed in a way that is logical and understandable, and is there some degree of analysis or commentary? YesAre the conclusions supported by the data in the results and the discussion? YesDaniel J. Kiely MDCM FRCSC MSc FACOGHôpital du Centre-de-la-Mauricie, Shawinigan, QC

Reviewer Expertise:

NA

No decision status is available
